# Supplementary material for: Altered glia-neuron communication in Alzheimer’s Disease affects WNT, p53, and NFkB Signaling determined by snRNA-seq
Source: Cell Commun Signal. 2024 Jun 7;22:317. doi: 10.1186/s12964-024-01686-8 (PMC11157763; doi:10.1186/s12964-024-01686-8)
Supplement: Supplementary file 4 — Supplementary Material 4 [file 12964_2024_1686_MOESM4_ESM.docx]

##
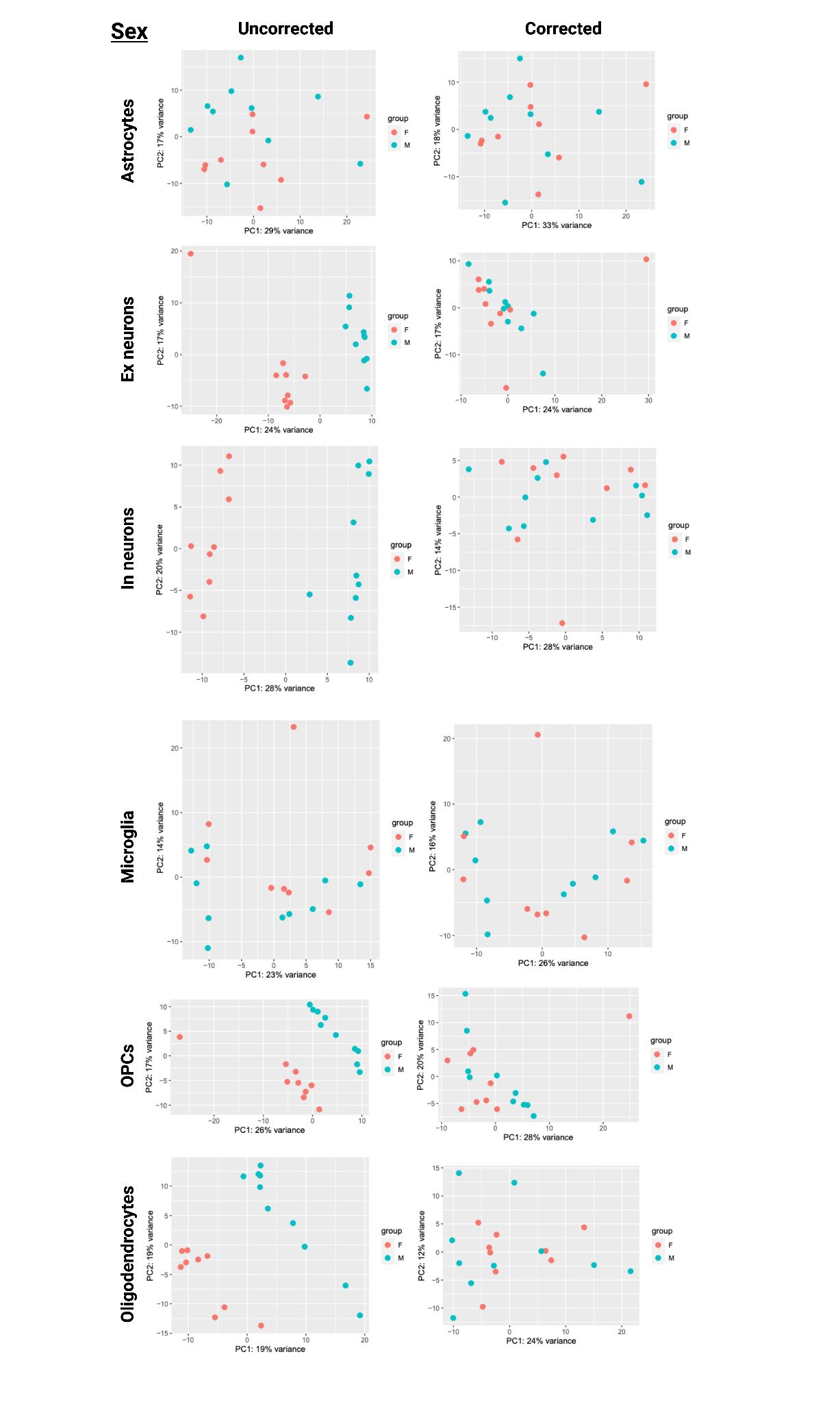
Figure S1: PCA plots before and after correction for patient sex in the Morabito et al. data. PCA plots colored by sex for all cell types of interest before adjustment for sex (left column) and after inclusion as a covariate (right column).

##

##

##

##

##

##

##

##

##

##

##

##

##

##

##

##

##

##

##

## Figure S2: PCA plots before and after correction for patient sex in the Lau et al. data. PCA plots colored by sex for all cell types of interest before adjustment for sex (left column) and after inclusion as a covariate (right column).

**
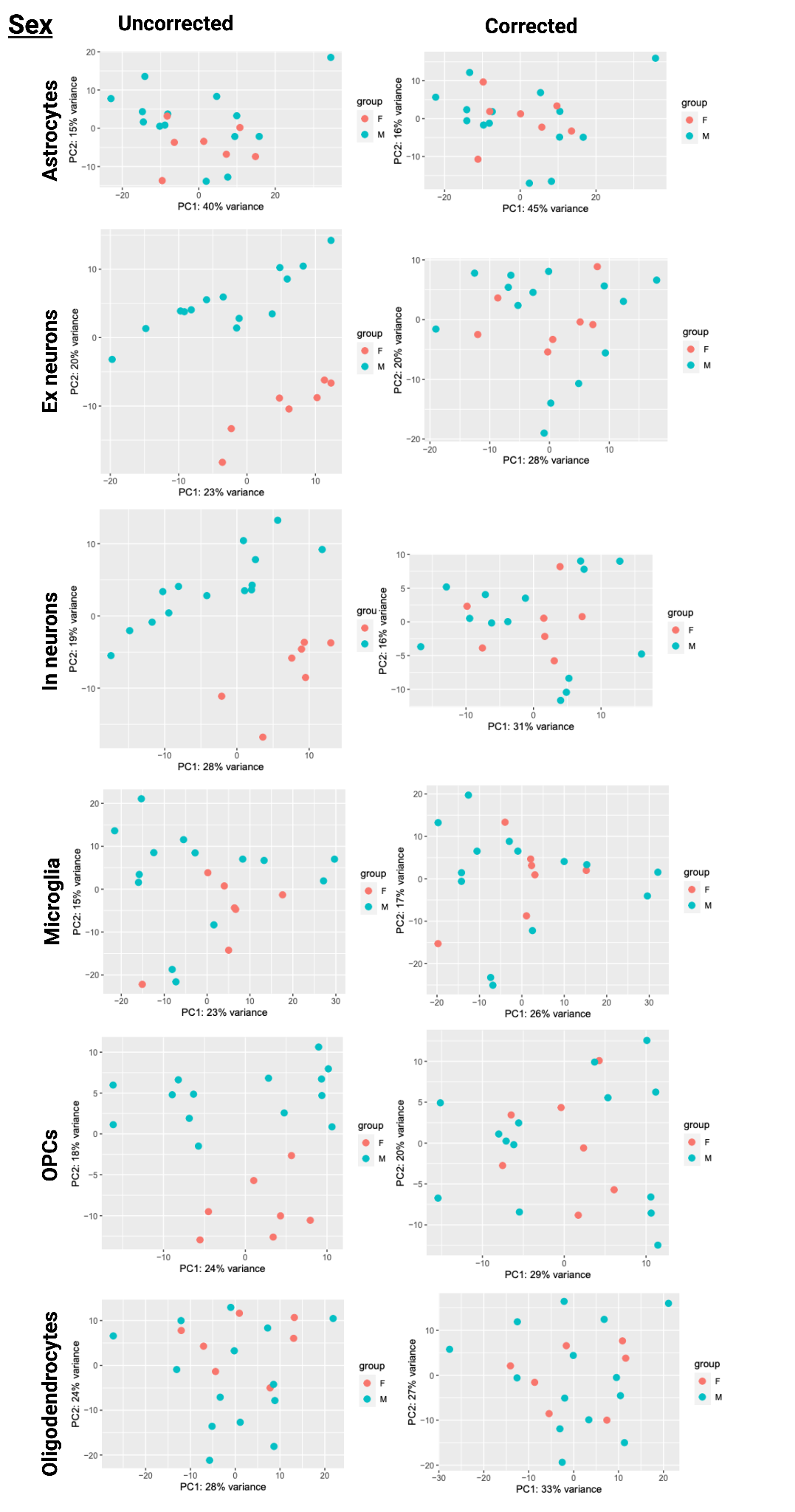
**

## Figure S3: Number of cell-cell communication interactions.

(**A**) Barplot showing the total number of predicted interactions across all cell types in AD. (**B**) Total number of interactions filtered by sender and receiver (excitatory and inhibitory neurons) cell types of interest, and (**C**) after prioritization using ligand activity and regulatory potential scores.


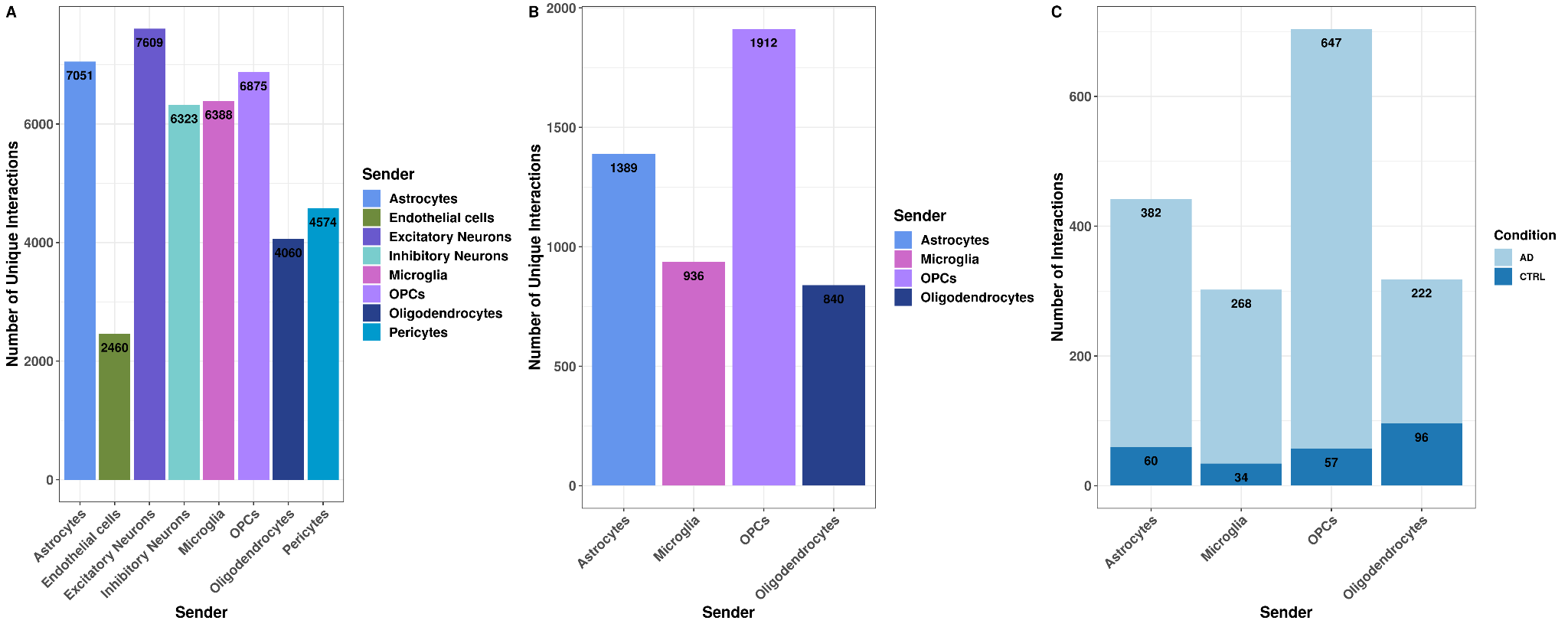


##

##

##

##

##

##

##

##

## Figure S4: Lau et al. cell type assignment and proportions

(**A**) Uniform Manifold Approximation and Projection (UMAP) of 32,003 nuclei colored by their assigned cell types. (**B**) Violin plot of canonical marker gene expression used for cell type assignment. Color represents cell type. (**C**) UMAP of integrated dataset split by condition (AD, CTRL). (**D**) Stacked barplot of cell type proportions across conditions (AD, CTRL).


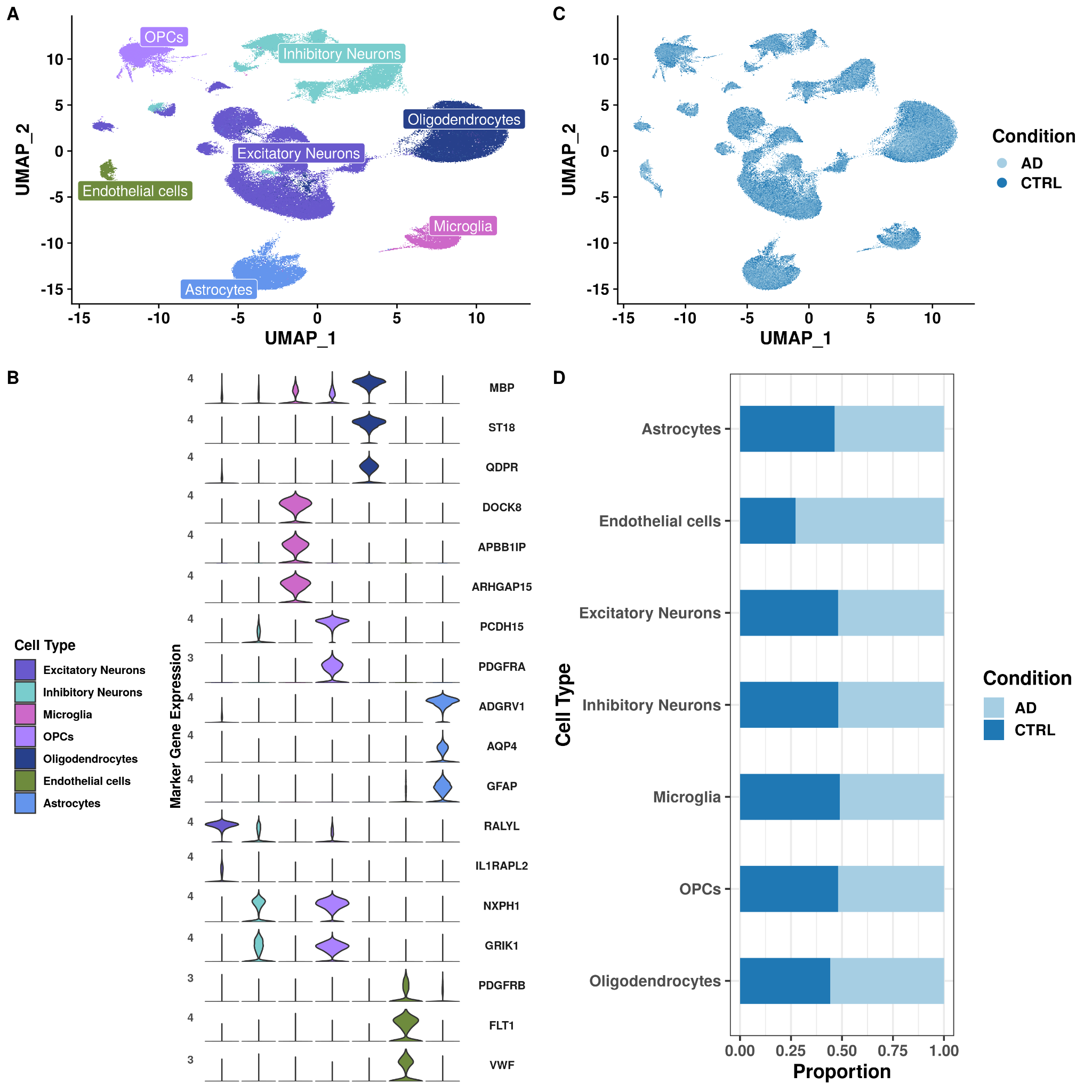


## Table S1: Morabito et al. Marker Genes

Table of marker genes in GSE174367. All genes are upregulated in their respective clusters and have an average log2fc > 0.2. We used the top 25 markers based on their adjusted p-value for each cluster for cell type assignment. [Table_S1.xlsx](https://docs.google.com/spreadsheets/d/1k1DPVzKf1VJTJ3V_lFFxgDfjEsQUFp06/edit?usp=sharing&ouid=104046041304042867219&rtpof=true&sd=true)

## Table S2: Lau et al. Marker Genes

Table of marker genes in GSE157827. All genes are upregulated in their respective clusters and have an average log2fc > 0.2. We used the top 25 markers based on their adjusted p-value for each cluster for cell type assignment. [Table_S2.xls](https://docs.google.com/spreadsheets/d/1D5QWSt5wBhUHCu2Mf-FXmlDRNmZkyckV/edit?usp=sharing&ouid=104046041304042867219&rtpof=true&sd=true)

## Table S3: Canonical marker genes Table of canonical marker genes used for cell type assignment using feature plots.

| **Canonical cell type markers** | **Cell type** |
| --- | --- |
| *PDGFRB* | Pericytes |
| *DOCK8*, *APBB1IP*, *ARHGAP15* | Microglia |
| *SNAP25*, *RBFOX1* | Neurons |
| *AQP4*, *GFAP*, *ADGRV1* | Astrocytes |
| *ST18*, *MBP*, *QDPR* | Oligodendrocytes |
| *PDGFRA*, *PCDH15* | OPCs |
| *FLT1*, *VWF* | Endothelial cells |

##

##

##

##

##

##

## Figure S5: Differential CCC analyses show low similarity of ligands, receptors, and targets across sender and receiver cell populations in Lau et al.

**(A)** Stacked barplot of the number of prioritized interactions where excitatory or inhibitory neurons are the receiver. **(B)** Jaccard Similarity Index (JI) between excitatory and inhibitory neurons of receptors and targets. JI between sender cell type **(C)** ligands, **(D)** receptors, and **(E)** targets for inhibitory neurons**.** JI between sender cell type **(F)** ligands, **(G)** receptors, **(H)** targets for excitatory neurons.


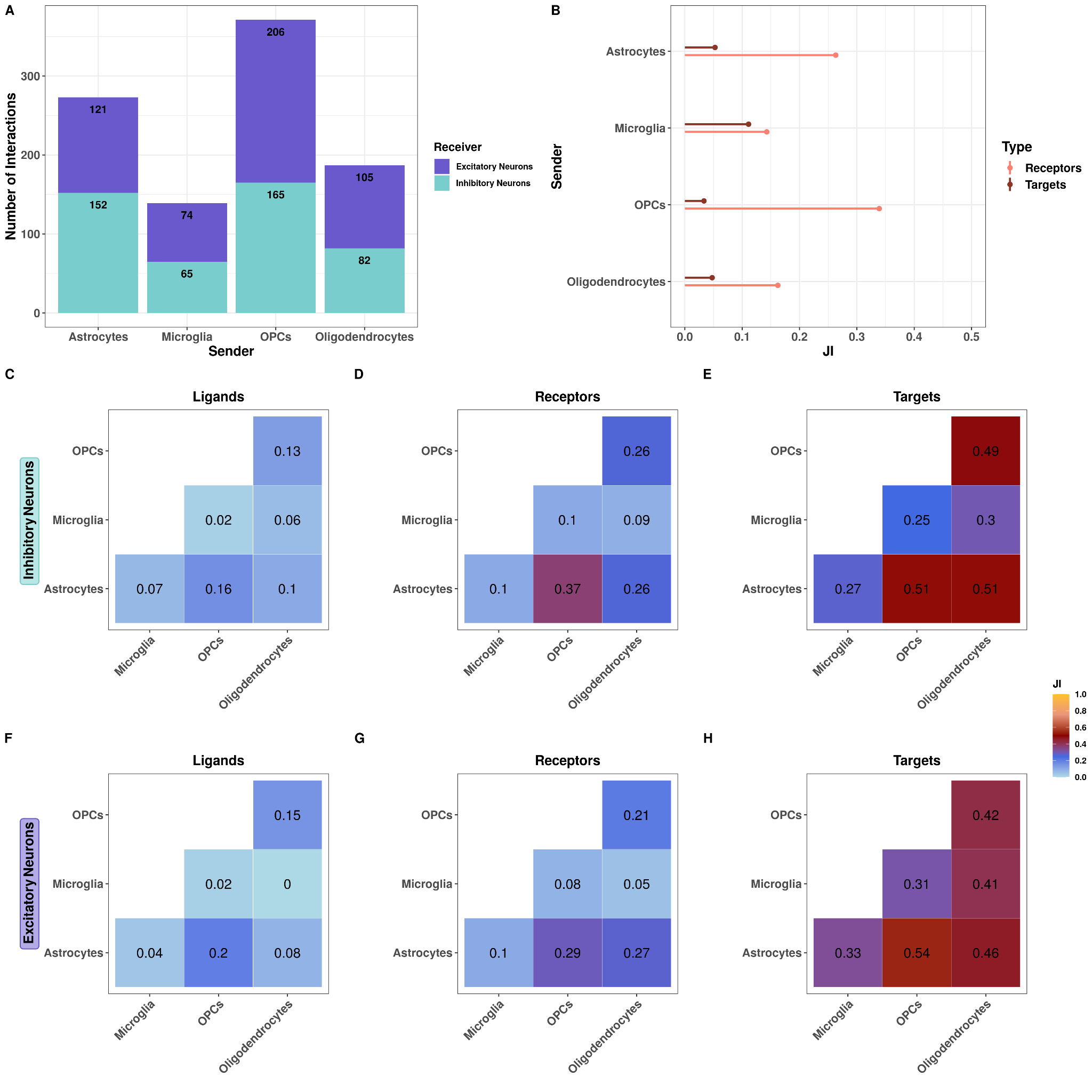


## Figure S6: Sadick et al. cell type assignment and proportions

(**A**) Uniform Manifold Approximation and Projection (UMAP) of over 100,000 nuclei colored by their assigned cell types. (**B**) Violin plot of canonical marker gene expression used for cell type assignment. Color represents cell type. (**C**) UMAP of integrated dataset split by condition (AD, CTRL). (**D**) Stacked barplot of cell type proportions across conditions (AD, CTRL).**
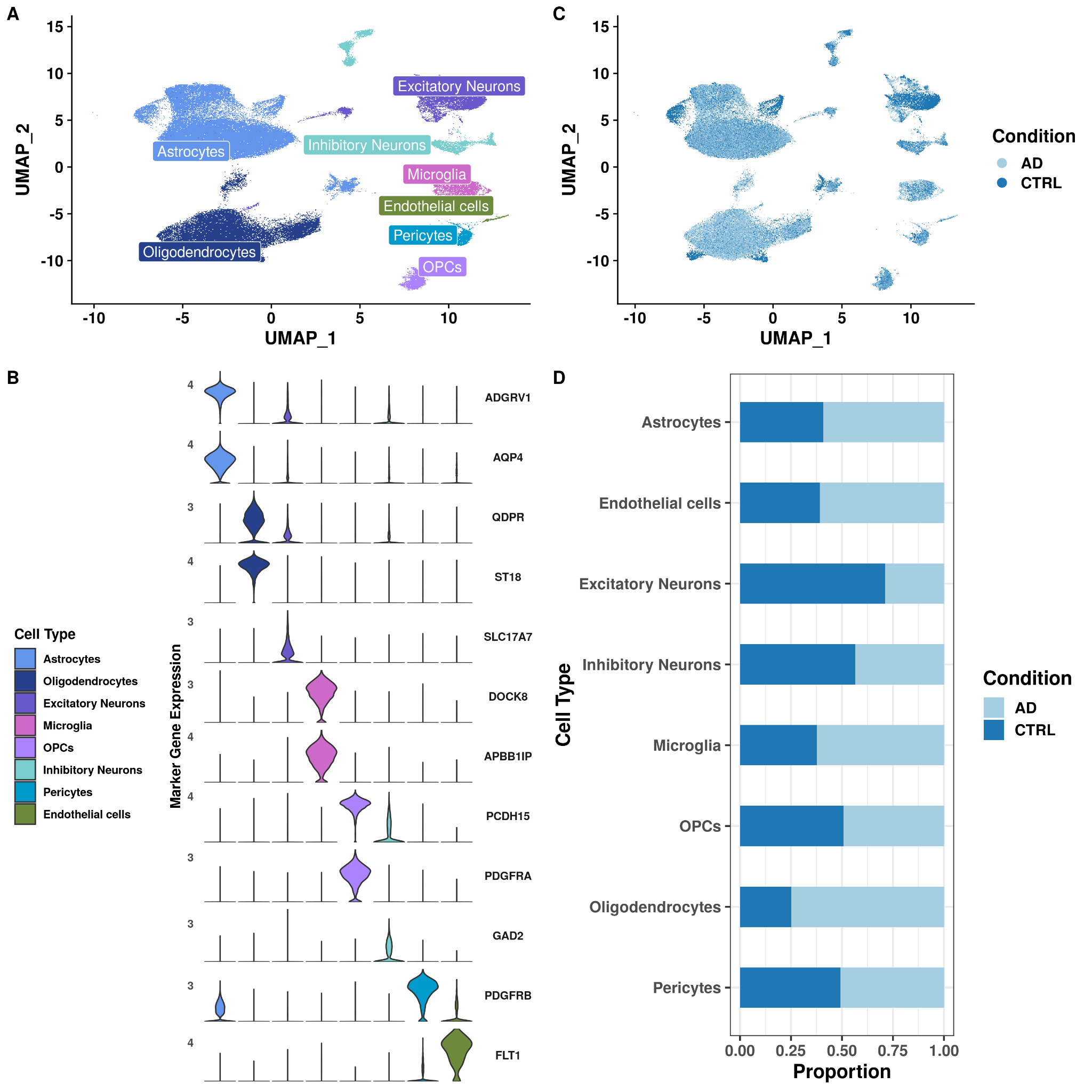
**

## Figure S7: Ligand-receptor and LRT overlap across all three snRNA-seq datasets

## (A) Upset plot of the overlapping ligand-receptor pairs (B) and LRTs. (C) Alluvial plot of the ligand-receptor pairs shared by all three datasets colored by sender cell type.
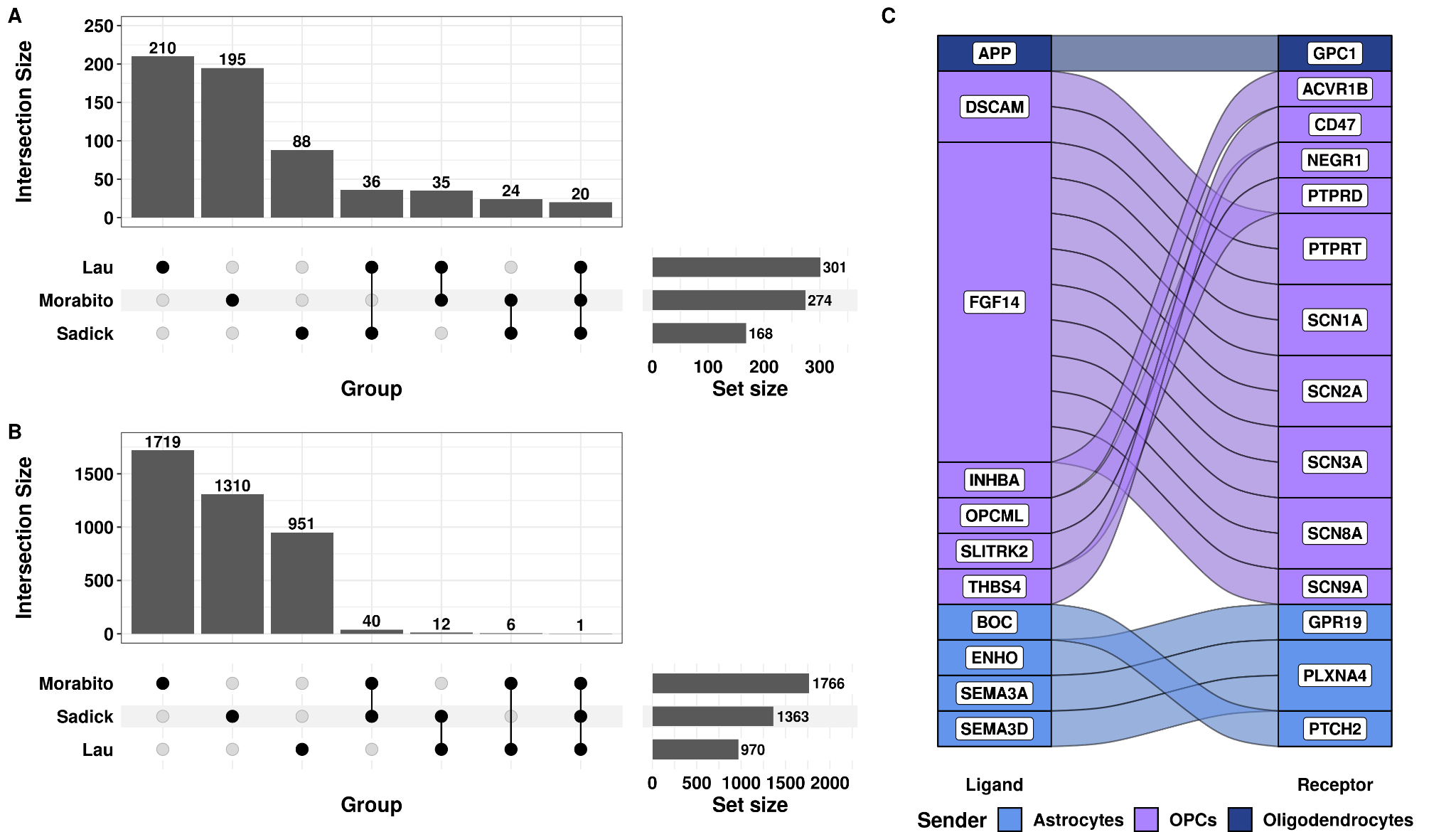


## Figure S8: Ligand-receptor pairs that overlap across two independent datasets. (A) Overlapping ligand-receptor pairs that were upregulated in AD. (B) Overlapping ligand-receptor pairs that were upregulated in control. Colored by sender cell type. Grey indicates the involvement of more than one sender.

##
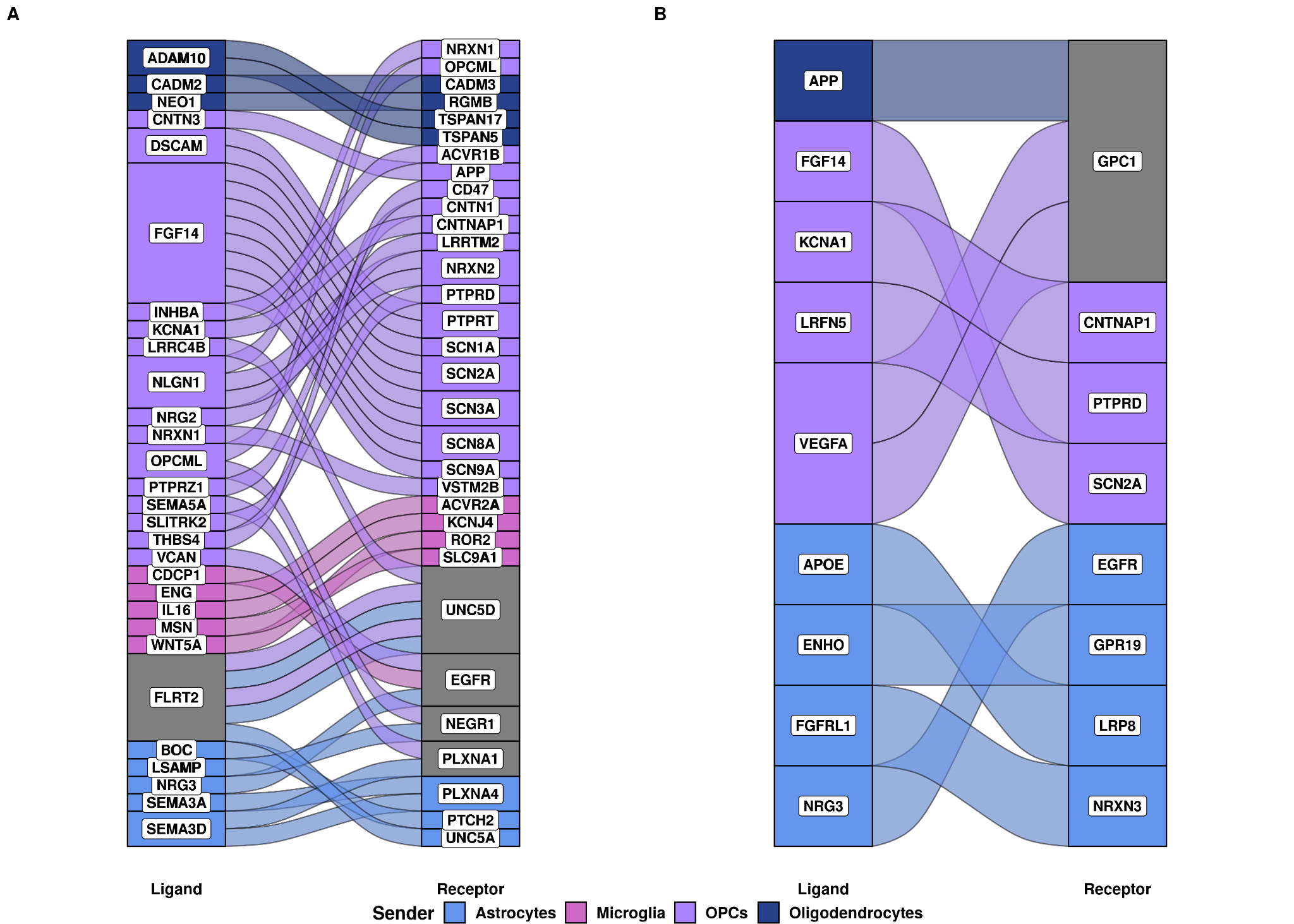


##

## Table S4: Target genes of ligand-receptor pairs that overlap across datasets

Table of 51 ligand-receptor pairs that overlap across datasets. The table includes the ligand-receptor pair, the sender and receiver cell type associated with the pair, as well as the target genes annotated by dataset of origin. [Table_S4.xlsx](https://docs.google.com/spreadsheets/d/1_za87zrXkhvuFVrgbA-RsUC13fNRs555/edit?usp=sharing&ouid=104046041304042867219&rtpof=true&sd=true)

## Figure S9: Alluvial plot of high-confidence LRTs between Morabito and Lau before using patient sex as a covariate.

##
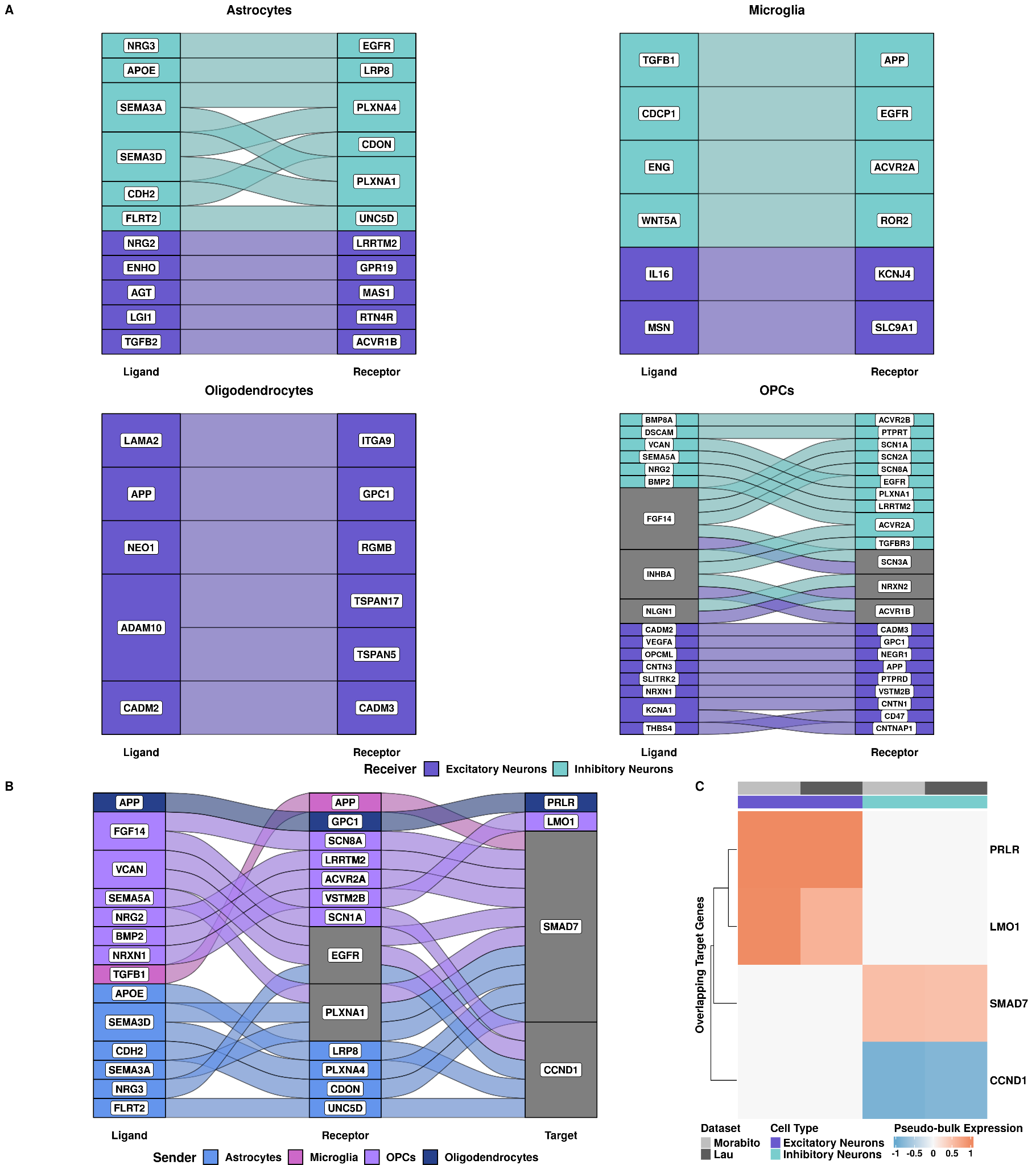


## 
